# Optimised paediatric antiretroviral treatment to achieve the 95-95-95 goals

**DOI:** 10.4102/sajhivmed.v22i1.1278

**Published:** 2021-09-01

**Authors:** Moherndran Archary, Riana van Zyl, Nosisa Sipambo, Gillian Sorour

**Affiliations:** 1 Department of Paediatrics and Child Health, College of Health Sciences, University of KwaZulu-Natal, Durban, South Africa; 2 Department of Paediatrics, King Edward VIII Hospital, Durban, South Africa; 3 Department of Paediatrics and Child Health, University of the Free State, Bloemfontein, South Africa; 4 Department of Paediatrics and Child Health, University of the Witwatersrand, Johannesburg, South Africa

**Keywords:** paediatric, HIV/AIDS, ART, 95-95-95 goals, PMTCT

## Abstract

While the progress towards reaching the UNAIDS 95-95-95 targets in South African adults seems promising, the progress in the paediatric population is lagging far behind; only 79% percent of children living with HIV know their status. Of these, only 47% are on treatment, and a mere 34% of those are virally suppressed. Thus, virological suppression has been attained in only 13% of children living with HIV in South Africa. Multiple factors contribute to the high treatment failure rate, one of them being a lack of paediatric-friendly antiretroviral treatment (ART) formulations. For example, the Lopinavir/ritonavir syrup, which is the current mainstay of ART for young children, has an extremely unpleasant taste, contributing to the poor tolerability and lack of adherence by children using the formulation. Furthermore, the lack of appropriate formulations limits the optimisation of regimens, especially for young children and those who cannot swallow tablets. Switching from syrups to dispersible tablets will improve ease of administration and adherence and result in cost-saving. Despite the approval of simplified paediatric-friendly formulations internationally, including other sub-Saharan African countries, unnecessary delays are experienced in South Africa. Clinician groups and community organisations must speak up and demand that approvals be expedited to ensure the delivery of life-changing and life-saving formulations to our patients as a matter of urgency.

According to the latest global Joint United Nations Programme on HIV and AIDS (UNAIDS) estimates, 1.8 million children live with HIV worldwide, with 150 000 new infections in children aged 0–14 years contributing to 9% of the overall new infections in 2019. Of these new infections, 84% occurred in sub-Saharan Africa, with around 95 000 HIV-related deaths in children reported in 2019.^1^ While South Africa’s prevention of mother-to-child transmission programme has been successful in decreasing the rate of vertical transmission of HIV to 3% (from 16% in 2010), paediatric HIV treatment programmes have not been as successful.^2^

In 2019, HIV-related deaths in South African children declined to 4100, and the number of children living with HIV remained more or less stable at 340 000.^1^ As part of working towards ending the HIV pandemic by 2030, attaining and maintaining virological suppression is critical. The current South African statistics in the overall population in terms of the UNAIDS 95-95-95 targets (diagnosis of 95% of all people living with HIV, achieving 95% on antiretroviral treatment [ART] among those diagnosed and 95% virally suppressed among those being treated) are 92-75-92.^1^ In the paediatric population, however, the progress towards meeting the treatment cascade goals is lagging far behind. Only 79% of children living with HIV know their status. Of these, only 47% are on treatment, and a mere 34% of those on treatment are virally suppressed.^1^

Treatment failure is multifactorial, but suboptimal adherence remains the most significant contributing factor. One of the reasons for non-adherence is the difficulty in obtaining treatment. The barriers to accessing treatment include physical challenges such as getting to clinics in remote areas, drug stock-outs and, recently, interrupted clinical services resulting from coronavirus disease 2019 (COVID-19) restrictions. Although the effect of lockdown had a more significant impact on HIV testing and the initiation of ART, the provision of ART was also affected.^3^ Disruptions to HIV programmes during the COVID-19 pandemic will have the most significant impact on HIV-related deaths. Based on modelling studies, the interruption of ART for 6 months in 50% of patients on ART will result in over 296 000 estimated deaths in sub-Saharan Africa annually.^4^

In the paediatric population, adherence is dependent on the motivation and commitment of the parent or caregiver. A lack of appropriate paediatric ART formulations adds to the burden of giving treatment to a child regularly enough to maintain high levels of adherence and obtain viral suppression. The classic ‘culprit’ is lopinavir/ritonavir (LPV/r) syrup: toddlers often refuse to take the treatment or vomit after the parent or caregiver administers the syrup because of the extremely unpleasant taste. In addition, the lack of fixed-dose combinations (FDC) for children results in complicated dosing regimens that negatively affect long-term adherence and retention in care.

## Supporting the move to simplified paediatric-friendly formulations

**FIGURE 1:**
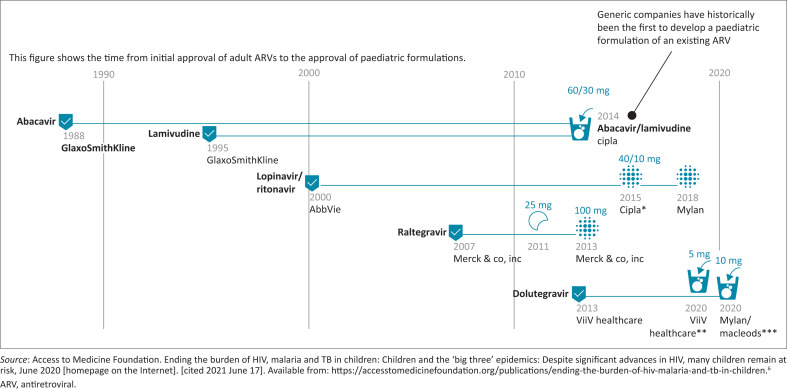
Timelines for paediatric antiretroviral approvals are shortening, but unnecessary delays remain.

The shift away from multiple syrup formulations for children unable to swallow tablets, previously the mainstay of the paediatric HIV treatment regimens in South Africa, has been delayed compared to other sub-Saharan African countries. This has been because of the painstakingly slow regulatory approval of generic FDC formulations designed for children. For example, the United States Food and Drug Administration (USFDA) approved the generic FDC of abacavir (ABC) and lamivudine (3TC) (120/60 mg) in 2014 (see Figure 1).^5^ The South African product submission to the South African Health Products Regulatory Authority (SAHPRA), then called the Medicines Control Council, only followed in 2016. Approval, however, only occurred in 2021, 7 years later.

The impact of optimised paediatric formulations cannot be overemphasised. For example, an 11 kg infant on ABC and 3TC will require a 6 mL twice-daily dose of each syrup, or 24 mL per day, and a total monthly supply of 672 mL, compared to a child who is switched to an FDC of ABC/3TC (120/60 mg) dispersible tablet (DT). Switching to the FDC ABC/3TC requires two tablets daily, or a bottle of 56 pills per month.^7^ These changes impact the patient and caregiver acceptability and adherence as well as the health system costs, including cost savings on syringes and a decrease in the required storage space.

## Abacavir/lamivudine fixed-dose combination formulations

The FDC of ABC/3TC (600/300 mg) was registered for use in South Africa from 2012 and was included in the paediatric and adolescent dosing guidelines soon afterwards, facilitating a once-daily nucleoside/nucleotide reverse transcriptase inhibitor (NRTI) backbone in children above 25 kg.^8^ This film-coated tablet should be swallowed whole and not cut or crushed, preventing the use of the formulation in younger children.

The new dispersible scored ABC/3TC FDC (120/60 mg) allows for flexible dosing and administration. The formulation can be dissolved in liquid and administered, chewed or swallowed whole, allowing the use of the formulation from young infants to older children. The dose proportions align with the World Health Organization (WHO) dosage recommendations developed to facilitate once-daily dosing of the NRTI backbone in children over 4 weeks of age and weighing 3 kg. The scored DT allows dosing in increments of 60/30 mg, which, when cut in half, allows for dosing across a wide weight range from 3 kg to 25 kg. In addition, the child-friendly flavouring of the formulation adds to the appeal. In South Africa, two generic manufacturers have made this FDC available to patients in the public sector.^7^

## Dolutegravir

The basis for introducing integrase inhibitors into the adult guidelines was the higher genetic barrier to resistance, improved side-effect profile and viral suppression rate associated with dolutegravir (DTG). While evidence for a potential association with neural-tube defects (NTDs) and increased weight gain has slowed down the introduction of DTG, especially in women of child-bearing potential, the opening into the paediatric population has been delayed by the lack of appropriate formulations. The inclusion of adolescents early into studies such as IMPAACT P1093^9,10^ has allowed their concurrent access, if weighing over 30 kg, to the 50 mg formulation as per the adult formulations in the national guidelines.^8^

The initial registration trial for DTG included 35 mg and 25 mg doses for children weighing 20 kg – 30 kg and 15 kg – 20 kg, respectively, requiring two additional formulations of a 25 mg and 10 mg tablet. Further pharmacokinetic evaluation conducted as a substudy of the Odyssey trial evaluated the use of the 50 mg tablet in children between 20 kg and 30 kg.^11^ Acceptable pharmacokinetics and side-effect profile supported the USFDA registration of DTG 50 mg daily in children from 20 kg. In addition, the new dosage recommendation was added to the WHO and several national guidelines, including the South African national ART guidelines.^8^

To dose children weighing below 20 kg, a 5 mg dispersible tablet of DTG (DTG DT) was developed by the originator, ViiV. Pharmacokinetic evaluation as part of the P1093 and Odyssey trials again supported the registration of the new formulation by the USFDA and European Medicines Agency in 2020 starting at 4 weeks of age and 3 kg in weight.^12^ In addition, as part of ViiV’s commitment to providing early access to DTG in low- and middle-income countries (LMICs), a sharing of the technical specifications with generic manufacturers has allowed the development of a scored 10 mg DTG DT.^13^ This has allowed DTG to be the first-line ART of choice across the age spectrum from 4 weeks of age in the new consolidated WHO guidelines. Registration of this new dispersible formulation by SAHPRA is eagerly awaited and will allow inclusion in the South African guidelines. The approval of DTG DT will facilitate the move away from using LPV/r syrup formulations and their associated problems.

At a recent conference presentation of the results of the Odyssey trial, there was no significant difference in weight gain in participants on a DTG versus non-DTG-containing regimen.^14^ In addition, the frequency of metabolic adverse events was lower in the DTG arm. These findings are reassuring and further support the use of DTG-based regimens in children. Furthermore, with an increase in the available cohort data, the association of NTDs and the use of DTG in the first trimester has decreased. The additional data support the use of DTG in women of child-bearing potential and has implications for introducing DTG in adolescent girls.

Tuberculosis is a common coinfection in South Africa, especially in people living with HIV. Co-treatment with rifampicin (Rif)-containing tuberculosis treatment and DTG results in a significant decrease in the DTG plasma concentration. Data in the adult population have supported 50 mg twice daily while on Rif to counteract the increased hepatic metabolism. In addition, a substudy of the Odyssey trial provided supportive data for this strategy in adolescents receiving Rif and DTG 50 mg tablets.^15^ There is a lack of data on the pharmacokinetics of Rif and DTG DT; however, based on the data from adults and adolescents, the USFDA in the registration of DTG DT recommended twice-daily dosing of DTG DT in children receiving Rif. Further studies are required to confirm this strategy; however, international guidelines are likely to support this approach while awaiting more supporting data.

As other global medicine regulatory authorities have extensively reviewed the formulation, advocates for the right of children to have access to the best available treatment options, clinician groups and community organisations need to be more vocal in demanding that the approval of this formulation be expedited in South Africa.

## Future innovations for simplified paediatric-friendly formulations

Further simplification of both treatment and prophylactic regimens for children in the future is likely to positively impact the goal of eliminating mother-to-child transmission and achieving the 95-95-95 milestone.

The recent registration of long-acting injectable cabotegravir/rilpivirine (LA CAB/RPV) administered every 2 months in HIV-infected individuals over 18 years by the USFDA highlights the direction of future treatment simplification. Results from the Mocha trial will support the registration of LA CAB/RPV in adolescents between 12 and 18 years old. While the need for refrigeration during storage of LA CAB/RPV may limit the use in LMICs, it opens up new treatment options. This is because LA CAB/RPV is administered intramuscularly, where it forms a crystalline structure that slowly releases the active drug into the plasma. A planned study in protocol development (IMPAACT 2036) hopes to explore LA CAB/RPV in younger children 2–12 years of age and aims to start in 2022. As we move these formulations into younger patients, the injection volumes, injection site (gluteal vs lateral thigh), changes in weight between injections and changes in the absorption and metabolism will need further evaluation.

Other formulations and delivery mechanisms in development include long-acting oral and implantable formulations (e.g. islatravir and lenacapavir), long-acting broadly neutralising antibodies and microarray patches. Each of these formulations is likely to play a significant part in the future treatment and prevention options for children and adolescents.

The scope for simplifying paediatric ART regimens has vastly improved, with the potential for a once-a-day solid formulation regimen from 4 weeks of age. Robust advocacy from clinicians and the community is required to ensure that these life-changing formulations are made available to our patients as soon as possible.
